# The investigation of semantic memory deficit in chronic tinnitus: a behavioral report^☆^

**DOI:** 10.1016/j.bjorl.2018.11.003

**Published:** 2018-12-28

**Authors:** Maryam Karimi Boroujeni, Saeid Mahmoudian, Farnoush Jarollahi

**Affiliations:** a Iran University of Medical Sciences (IUMS), School of Rehabilitation Sciences, Department of Audiology, Tehran, Iran; b Iran University of Medical Sciences (IUMS), ENT and Head & Neck Research Center, Tehran, Iran

**Keywords:** Tinnitus, Semantics, Memory, Zumbido, Semântica, Memória

## Abstract

**Introduction:**

Tinnitus is a central auditory disorder in which different processing systems are involved as a network. One of these networks is memory. Previous studies have demonstrated some deficits in various types of memory in chronic tinnitus.

**Objectives:**

The main purpose of the present study was to investigate the semantic memory, which is not yet investigated in the tinnitus population.

**Methods:**

In this case–control study, 15 subjects with chronic tinnitus and 16 matched healthy controls were included. 40 semantically related and 40 semantically unrelated word pairs were presented to the participants in a counter-balanced fashion. They were asked to make decision about their semantic relatedness. Then the participants’ reaction times and the accuracy of responses were calculated.

**Results:**

Mean of reaction times were significantly longer in the tinnitus group (*M* = 1034 ms, SD = 0.31) compared to the control group (Mean = 1016 ms, SD = 0.13), *p* < 0.05. However, no significant difference was found for the mean percentage of correct responses between the two groups.

**Conclusion:**

The current study provided behavioral evidence that chronic tinnitus can affect the semantic memory. Such behavioral outcomes may provide new insights into more research activities in the field of electrophysiology and neuroimaging in the tinnitus population.

## Resumo

**Introdução:**

O zumbido é um distúrbio auditivo central, no qual diferentes sistemas de processamento estão envolvidos como em uma rede. Uma dessas redes é a memória. Estudos anteriores demonstraram alguns déficits em vários tipos de memória no zumbido crônico.

**Objetivos:**

Investigar a memória semântica, que ainda não foi investigada na população com zumbido.

**Método:**

Neste estudo de caso-controle, 15 indivíduos com zumbido crônico e 16 controles saudáveis pareados foram incluídos; 40 pares de palavras semanticamente relacionados e 40 semanticamente não relacionados foram apresentados aos participantes de forma contrabalançada. Eles foram instruídos a tomar decisões sobre sua relação semântica. Em seguida, os tempos de reação dos participantes e a precisão das respostas foram calculados.

**Resultados:**

A média dos tempos de reação foi significativamente maior no grupo com zumbido (M = 1,034 ms, DP = 0,31) em comparação ao grupo controle (média = 1016 ms, DP = 0,13), *p* < 0,05. Entretanto, nenhuma diferença significante foi encontrada para a porcentagem média de respostas corretas entre os dois grupos.

**Conclusão:**

O presente estudo forneceu evidências comportamentais de que o zumbido crônico pode afetar a memória semântica. Tais resultados comportamentais podem levar a novas percepções em mais atividades de pesquisa no campo da eletrofisiologia e neuroimagem na população com zumbido.

## Introduction

Tinnitus as an auditory phantom perception refers to the conscious experience of sound in the absence of any external acoustic stimuli.^1^, ^2^, ^3^, ^4^, ^5^ Being considered as a subjective perception, tinnitus can affect quality of life differently in people experiencing it.^6^ Whereas some tinnitus patients can cope with their tinnitus perfectly, others abysmally suffer from the consequences resulting from chronic tinnitus, such as anxiety, depression, sleep disturbance, emotional disorders, work impairment, and concentration problems. In severe conditions, maladapted coping behaviors may lead to suicide.^7^, ^8^, ^9^, ^10^

Such symptoms and problems refer to the involvement of non-auditory areas of the brain which work as a network alongside the auditory cortex, albeit, this co-existing mechanisms are still controversial.^11^, ^12^ Recent studies have revealed the involvement of non-auditory structures, most notably those involved in memory, cognitive, attentional, and emotional networks in chronic tinnitus.^1^, ^13^, ^14^

Specifically, the memory system as one of the components of this network plays a significant role in the awareness of tinnitus and tinnitus-related distress.^7^, ^11^ Previous studies have revealed that the hippocampus and parahippocampal areas were functionally altered in chronic tinnitus.^4^, ^11^, ^15^ These regions are crucial in short-term memory and auditory-verbal memory.^16^, ^17^ Such these abnormalities have been detected in the dorsolateral prefrontal cortex which is involved in working memory.^4^, ^18^, ^19^ Moreover, the amygdala and limbic system have also indicated an overlap between tinnitus network and the brain regions involved in auditory memory.^11^, ^15^, ^20^

Among different types of memory, the sensory memory defect in the central auditory pathway has been indexed by the reduction of amplitude and area under the curve of the Mismatch Negativity (MMN) response, an auditory ERP reflecting the neural basis of the auditory sensory memory, in tinnitus population.^21^ In addition, the results of Autobiographical Memory Test (AMT) in tinnitus sufferers showed difficulty in retrieving specific memories and longer retrieval latencies, with fewer specific memories to positive cue words in comparison with a normal population.^22^ Furthermore, as reported by Rossiter and colleagues, chronic tinnitus affected the auditory working memory, which reduced its capacity to store and retrieve information.^23^

Semantic memory is another aspect of memory that is the storehouse of a wide range of knowledge acquired through experience, which is not reviewed in tinnitus population yet.^20^, ^24^, ^25^ This kind of declarative memory encompasses all the information used in thoughts and language, such as beliefs, word meaning, all abstract concepts and associations between them.^26^, ^27^ Thus, semantic memory allows us to retrieve stored information that is used in thoughts and language.^24^, ^28^

Although the effects of tinnitus on aspects of memory such as the long term memory has confirmed, the semantic memory as a subset of the long term memory has not been specifically studied in tinnitus patients. Moreover, the overlap between brain regions involved in chronic tinnitus and the anatomical regions specialized for the different aspects of semantic processing impelled us to investigate the interplay of tinnitus and semantic memory.^13^, ^14^ However, one previous study has demonstrated that the progressive erosion of semantic memory makes this population more liable to develop tinnitus.^29^ Accordingly, the present study has assumed that semantic memory is affected in tinnitus patients. Since there are still no studies concerning the relation between semantic memory and chronic tinnitus, focusing on this subject can provide a new insight into the research activities and medical-rehabilitation approaches that can be used for tinnitus population. Therefore, the present study aimed to investigate semantic memory in tinnitus patients with regard to the basic assumption that the tasks requiring memory can be influenced by troublesome tinnitus.

## Methods

### Participants

The experimental groups consisted of 15 tinnitus subjects experiencing chronic tinnitus for more than 6 months (mean age = 37.67, SD = ±11.47, range: 23–55 years; 5 females) and 16 healthy subjects as a control group, the two groups were matched for sex and age. None of these subjects had a history of neurological, mental, or otological diseases, head trauma, and alcohol/drug abuse. All subjects had the behavioral pure tone audiometry threshold levels of 25 dB HL or less at octave frequencies of 250–2000 Hz and not more than 40 dB HL in frequencies of 4000 and 8000 Hz. All participants were monolingual, native Persian speakers. Having normal cognition status was another inclusion criteria measured using Persian version of the Mini-Mental State Exam (MMSE).^30^ All subjects were also given the validated Persian version of Hospital Anxiety and Depression Scale (HADS) and those with a score of 21 or less (less than 11 for either depression and anxiety subscales, referring not to have depression or anxiety disorders) were included.^31^, ^32^ Furthermore, the Persian version of the Tinnitus Handicapped Inventory (THI) and Tinnitus Questionnaire (TQ) were filled by tinnitus patients.^33^, ^34^ The tinnitus subjects who had scores more than 60% in TQ and 58 in THI were enrolled in the study. In addition, the educational level for participation was at least diploma, as a reliable sign of general cognitive abilities.

### Stimuli

Stimuli included 40 semantically related and 40 semantically unrelated prime-target word pairs, which are known as the semantic priming paradigm.^35^, ^36^ Across related and unrelated conditions, the target words and the prime words were separately matched for length and frequency. The relatedness proportion as another methodological consideration used to provide semantic priming paradigm was 0.5.^37^ The words selection and matching in the form of semantically related and unrelated pairs were controlled under two linguistic experts’ supervision. This paradigm was validated (CVR score was more than 62% for all word pairs; CVI in the total score = 0.93) and its reliability was determined (*α* = 0.93 for semantically related pairs; *α* = 0.92 for semantically unrelated pairs; and *α* = 0.94 for all items). These pairs of words were presented to the participants in a counter-balanced fashion.

### Procedure

In a quiet room, the participants heard the word pairs through loudspeakers in free field in the following order: (a) the presentation of the prime word; (b) a silence gap for 1150 ms; (c) the presentation of the target word; and (d) a silence gap for 3000 ms for participants’ response. Then the participants were told that they were going to hear two words in each trial. At the prompt, they should make a decision whether there is a semantic relationship between these two words or not. To show their semantic judgment, they would press left-click on the mouse for the semantically related pairs and right-click for the semantically unrelated ones as soon as they hear the second word. It is necessary to mention that the recorded words were given to the participants using presentation software. The intensity level of stimuli was typically adjusted to the individual's Most Comfortable Level (MCL).

All the procedures performed in this study were in accordance with Ethics Committee of Iran University of medical sciences and its later amendments or comparable ethical standards. All participants had been given a written informed consent for their participation. After completing their participation, patients who declared a need for getting more help were referred to the tinnitus clinic to benefit from the treatment programs available in our country.

### Behavioral data collection and analysis

During the experimental task, the Reaction Times (RTs) as an indicator of processing efficiency were obtained for semantically related and unrelated pairs separately. To this end, the RTs were calculated from the onset of target word until the mouse button was pressed by participants. According to a defined default in the presentation software, if participants pressed the mouse button before the offset of the target word, the presented item would not be accounted as a response. Lastly, mean percentages of correct responses also obtained for the related and unrelated condition.

### Statistical analyses

Firstly, normality of distributions of the data was examined by calculating the standardized skewness and kurtosis index. Values varied from −2 to 2, indicating that the data distributions did not differ significantly from normality. Thus, an independent *t*-test (significance level of 0.05) was used to compare the RTs between the tinnitus sufferers and the control group, both for semantically related and unrelated pairs. Moreover for the accuracy of responses, the percentage of correct responses obtained through each stimulus type (the related and unrelated pairs) was also analyzed by independent *t*-test. The Statistical Package for Social Science (SPSS V.16; Chicago, United States) was used to perform all statistical analyses.

## Results

As previously mentioned, the current study consisted of 15 tinnitus patients and 16 healthy subjects having been matched in aspect of age, gender, HADS and MMSE score (*p* > 0.05). In addition, the results of TQ and THI questionnaires represented the tinnitus subjects as a homogeneous group. A summary of participant characteristics is given in Table 1.

**Table 1:** Summary of participant demographics and the results of used questionnaires.

Variables	Studied groups	
	Normal	Tinnitus
*Sex*		
Female	7 (43.75%)	5 (33.33%)
Male	9 (56.25%)	10 (66.66%)

Age (mean ± SD)	38.25 ± 7.98	37.67 ± 11.47
MMSE score (mean ± SD)	29.90 ± 0.29	29.86 ± 0.35
HADS score (mean ± SD)	7.14 ± 2.19	9.02 ± 1.81
TQ score (mean ± SD)	–	70.93 ± 6.18
THI score (mean ± SD)	–	68.53 ± 8.6

In the following, an independent *t*-test was conducted to compare the RTs between the two studied groups. As shown in Table 2, for the semantically related prime-target words, the mean of RTs significantly differed between tinnitus group and control group (*p* < 0.05). Furthermore for the semantically unrelated pairs, a significant difference was seen in the mean of RTs in tinnitus subjects compared to the control group (*p* < 0.05).

**Table 2:** Mean of reaction times (ms).

Stimulus type	Studied groups		*p*-Value
	Normal (mean ± SE)	Tinnitus (mean ± SE)	
Semantically related word pairs	1016 ± 0.13	1034 ± 0.31	0.046
Semantically unrelated word pairs	1029 ± 0.20	1051 ± 0.36	0.041

The responses’ accuracy was also compared by independent *t*-test. The high correct-response rates revealed that the two groups paid attention to stimuli and task and did not differ significantly in making a judgment about the semantic relatedness of the word pairs. No significant differences were seen between the two studied groups for all the word pairs (*p* > 0.05). Table 3 represents mean percentage of correct responses.

**Table 3:** Mean percentages of correct responses (%).

Stimulus type	Studied groups		*p*-Value
	Normal	Tinnitus	
Semantically related word pairs	98.90	98.0	0.215
Semantically unrelated word pairs	98.28	98.16	0.880

## Discussion

The main aim of this study was to investigate semantic memory in patients with chronic tinnitus. To this end, the semantic priming paradigm was used as a behavioral tool for studying semantic memory. For that, the accuracy of responses and the speed in the identification of semantic relationship which is indexed by the RT were calculated.^38^ As expected, not only the control group but also the tinnitus subjects had shorter RTs in response to the semantically related pairs rather than the unrelated ones. This result corresponded closely to other studies used the priming paradigm. According to distributed models, if the prime and the target are semantically related, the target word will be recognized more rapidly.^36^, ^39^, ^40^ Indeed, since semantic memory is formed from a set of nodes that are interconnected based on their semantic similarities, the processing of semantically related pairs would be easier and faster.^27^, ^28^, ^41^

Regarding the main purpose of the present study, the results of comparison of RTs between the studied groups should be taken into consideration. This experiment demonstrated that the tinnitus subjects significantly responded to our stimuli later than the control group, whether they are semantically unrelated pairs or not. This increment in RTs can be an indication of the deficit in semantic memory. In other words, the more difficulty in semantic processing, the more increase in the RTs. It seems that the overlap of regions involved in tinnitus and semantic processing leads to defective semantic processing in tinnitus sufferers. According to Martin, 2001, the prefrontal cortex is one of the neural structures that has an important role in retrieving, maintaining and selecting semantic information.^24^ The evidence has shown that the prefrontal cortex changes structurally and functionally in chronic tinnitus.^13^ Furthermore, the involvement of the middle temporal gyrus in tinnitus network^42^, ^43^, ^44^, ^45^ and also in long-term storage of lexical representation^28^ supports our results. Moreover, MRI data from semantic dementia patients has revealed an increase in the gray matter of the posterior superior temporal gyrus and sulcus. These regions contain the association auditory córtex.^29^ Furthermore, the limbic system as another common cortical region plays a major role in both semantic processing and chronic tinnitus.^20^, ^46^ Indeed, the damage involving such areas in tinnitus subjects can make this population more susceptible to have semantic memory deficit.

On the other hand, the longer RTs in tinnitus subjects can be a result of their poor performance in the process of integration of the target word with the prime word. As mentioned above, the semantic prime paradigm was used in this study. According to semantic processing, the presentation of the prime word leads to activation of the long-term semantic memory. When the prime word is retrieved, it should be held in short-term memory so that the target word can be integrated with it.^28^, ^40^ The holding information in short-term memory is one of the responsibilities of working memory.^47^ Previous studies have demonstrated that chronic tinnitus has an effect on the working memory. Thus, it is expected that the tinnitus subjects perform poorly in this task.

Considering the high percentage of correct responses, it appeared that all subjects were attending to the stimuli and task. Accordingly, it can be concluded that the longer RTs in chronic tinnitus patients have not been due to their inattention and distraction.

On the other hand, the tinnitus patients had been homogeneous lyselected from the aspect of mental state, tinnitus loudness, and tinnitus annoyance. Moreover, the results of the HADS questionnaire revealed no depression in the tinnitus group. As depression can be associated with disrupted memory for positive materials and enhanced memory for negative materials,^48^ the lack of depression can show that our findings are exclusively due to tinnitus, not depression. Accordingly, in the tinnitus management and rehabilitation, the experts can capitalize on some special programs targeted at the different aspects of memory network. Moreover, since the human's beliefs and thoughts are stored in semantic memory, the Cognitive Behavioral Therapy (CBT) can lay a fertile ground for adding specific programs to change the negative plasticity in tinnitus patients and improve their performance in memory tasks.

## Conclusion

The current study indicated a possible deficit in semantic memory in tinnitus subjects. In this regard, tinnitus sufferers revealed poor performance in using meaningful context to activate semantic memory. The longer RTs were a confirmation of this report. Although the study had reached its purpose, it is suggested that the experiment be conducted with a larger group. To confirm the results of RTs, the structural and functional studies can be used for investigating semantic processing in chronic tinnitus.

## Conflicts of interest

The authors declare no conflicts of interest.

## References

[1] Baizer J.S., Manohar S., Paolone N.A., Weinstock N., Salvi R.J. (2012). Understanding tinnitus: the dorsal cochlear nucleus, organization and plasticity. Brain Res.

[2] Chen Y.C., Feng Y., Xu J.J., Mao C.N., XiaW, Ren J. (2016). Disrupted brain functional network architecture in chronic tinnitus patients. Front Aging Neurosci.

[3] Jastreboff P.J., Sasaki C.T. (1994). An animal model of tinnitus: a decade of development. Am J Otol.

[4] Hong S.K., Park S., Ahn M.-H., Min B.-K. (2016). Top-down and bottom-up neurodynamic evidence in patients with tinnitus. Hear Res.

[5] Onishi E.T., Coelho C.C.B., Oiticica J., Figueirdo R.R., Guimarães R.C.C., Sanchez T.G. (2017). Tinnitus and sound intolerance: evidence and experience of a Brazilian group. Braz J Otorhinolaryngol.

[6] Roberts L.E., Eggermont J.J., Caspary D.M., Shore S.E., Melcher J.R., Kaltenbach J.A. (2010). Ringing ears: the neuroscience of tinnitus. J Neurosci.

[7] De Ridder D., Elgoyhen A.B., Romo R., Langguth B. (2011). Phantom percepts: tinnitus and pain as persisting aversive memory networks. Proc Natl Acad Sci U S A.

[8] Vanneste S., Plazier M., der Loo Ev, de Heyning P.V., Congedo M., De Ridder D. (2010). The neural correlates of tinnitus-related distress. Neuroimage.

[9] Erlandsson S.I., Holgers K.M. (2001). The impact of perceived tinnitus severity on health-related quality of life with aspects of gender. Noise Health.

[10] Scott B., Lindberg P. (2000). Psychological profile and somatic complaints between help-seeking and non-help-seeking tinnitus subjects. Psychosomatics.

[11] Langguth B., Schecklmann M., Lehner A., Landgrebe M., Peoppl T.B., Kreuzer P.M. (2012). Neuroimaging and neuromodulation: complementary approaches for identifying the neuronal correlates of tinnitus. Front Syst Neurosci.

[12] Vanneste S., Joos K., Langguth B., To W.T., De Ridder D. (2014). Neuronal correlates of maladaptive coping: an EEG-study in tinnitus patients. PLOS ONE.

[13] Laureano M.R., Onishi E.T., Bressan R.A., Castiglioni M.L., Batista I.R., Reis M.A. (2014). Memory networks in tinnitus: a functional brain image study. PLOS ONE.

[14] Langguth B., Elgoyhen A.B. (2012). Current pharmacological treatments for tinnitus. Expert Opin Pharmacother.

[15] Schmidt S.A., Carpenter-Thompson J., Husain F.T. (2017). Connectivity of precuneus to the default mode and dorsal attention networks: a possible invariant marker of long-term tinnitus. Neuroimage Clin.

[16] Sauseng P., Klimesch W. (2008). What does phase information of oscillatory brain activity tell us about cognitive processes?. Neurosci Biobehav Rev.

[17] Squire L.R. (1992). Memory and the hippocampus: a synthesis from findings with rats, monkeys, and humans. Psychol Rev.

[18] Bechara A., Martin E.M. (2004). Impaired decision making related to working memory deficits in individuals with substance addictions. Neuropsychology.

[19] Baddeley A. (1992). Working memory. Science.

[20] Binder J.R., Desai R.H., Graves W.W., Conant L.L. (2009). Where is the semantic system? A critical review and meta-analysis of 120 functional neuroimaging studies. Cereb Cortex.

[21] Mahmoudian S., Farhadi M., Najafi-Koopaie M., Darestani-Farahani E., Mohebbi M., Dengler R. (2013). Central auditory processing during chronic tinnitus as indexed by topographical maps of the mismatch negativity obtained with the multi-feature paradigm. Brain Res.

[22] Andersson G., Hesser H., Cima R.F., Weise C. (2013). Autobiographical memory specificity in patients with tinnitus versus patients with depression and normal controls. Cogn Behav Ther.

[23] Rossiter S., Stevens C., Walker G. (2006). Tinnitus and its effect on working memory and attention. J Speech Lang Hear Res.

[24] Martin A., Chao L.L. (2001). Semantic memory and the brain: structure and processes. Curr Opin Neurobiol.

[25] Patterson K., Nestor P.J., Rogers T.T. (2007). Where do you know what you know? The representation of semantic knowledge in the human brain. Nat Rev Neurosci.

[26] Binder J.R., Desai R.H. (2011). The neurobiology of semantic memory. Trends Cogn Sci.

[27] Kutas M., Federmeier K.D. (2000). Electrophysiology reveals semantic memory use in language comprehension. Trends Cogn Sci.

[28] Lau E.F., Phillips C., Poeppel D. (2008). A cortical network for semantics:(de) constructing the N400. Nat Rev Neurosci.

[29] Mahoney C.J., Rohrer J.D., Goll J.C., Fox N.C., Rossor M.N., Warren J.D. (2011). Structural neuroanatomy of tinnitus and hyperacusis in semantic dementia. J Neurol Neurosurg Psychiatry.

[30] Ansari N.N., Naghdi S., Hasson S., Valizadeh L., Jalaie S. (2010). Validation of a Mini-Mental State Examination (MMSE) for the Persian population: a pilot study. Appl Neuropsychol.

[31] Montazeri A., Vahdaninia M., Ebrahimi M., Jarvandi S. (2003). The Hospital Anxiety and Depression Scale (HADS): translation and validation study of the Iranian version. Health Qual Life Outcomes.

[32] Snaith R.P. (2003). The hospital anxiety and depression scale. Health Qual Life Outcomes.

[33] Mahmoudian S., Shahmiri E., Rouzbahani M., Jafari Z., Keyhani M.R., Rahimi F. (2011). Persian language version of the “Tinnitus Handicap Inventory”: translation, standardization, validity and reliability. Int Tinnitus J.

[34] Daneshi A., Mahmoudian S., Farhadi M., Hasanzadeh S., Ghalebaghi B. (2005). Auditory electrical tinnitus suppression in patients with and without implants. Int Tinnitus J.

[35] Duncan C.C., Barry R.J., Connolly J.F., Fischer C., Michie P.T., Naatanen R. (2009). Event-related potentials in clinical research: guidelines for eliciting, recording, and quantifying mismatch negativity, P300, and N400. Clin Neurophysiol.

[36] Matsumoto A., Iidaka T., Haneda K., Okada T., Sadato N. (2005). Linking semantic priming effect in functional MRI and event-related potentials. Neuroimage.

[37] Altarriba J., Basnight D.M. (2007). Methodological considerations in performing semantic-and translation-priming experiments across languages. Behav Res Methods.

[38] Calvo M.G., Avero P. (2009). Reaction time normative data for the IAPS as a function of display time, gender, and picture content. Behav Res Methods.

[39] Rossell S.L., Price C.J., Nobre A.C. (2003). The anatomy and time course of semantic priming investigated by fMRI and ERPs. Neuropsychologia.

[40] Moldovan C.D., Ferré P., Demestre J., Sánchez-Casas R. (2015). Semantic similarity: normative ratings for 185 Spanish noun triplets. Behav Res Methods.

[41] Kenett Y.N., Kenett D.Y., Ben-Jacob E., Faust M. (2011). Global and local features of semantic networks: evidence from the Hebrew mental lexicon. PLoS ONE.

[42] Vanneste S., De Ridder D. (2012). The auditory and non-auditory brain areas involved in tinnitus. An emergent property of multiple parallel overlapping subnetworks. Front Syst Neurosci.

[43] Farhadi M., Mahmoudian S., Saddadi F., Karimian A.R., Mirzaee M., Ahmadizadeh M. (2010). Functional brain abnormalities localized in 55 chronic tinnitus patients: fusion of SPECT coincidence imaging and MRI. J Cereb Blood Flow Metab.

[44] Shulman A., Goldstein B., Strashun A.M. (2007). Central nervous system neurodegeneration and tinnitus: a clinical experience. Int Tinnitus J.

[45] Chen Y.C., Xia W., Chen H., Feng Y., Xu J.J., Gu J.P. (2017). Tinnitus distress is linked to enhanced resting-state functional connectivity from the limbic system to the auditory cortex. Hum Brain Mapp.

[46] Lockwood A.H., Salvi R.J., Coad M., Towsley M., Wack D., Murphy B. (1998). The functional neuroanatomy of tinnitus: evidence for limbic system links and neural plasticity. Neurology.

[47] Cowan N. (2008). What are the differences between long-term, short-term, and working memory?. Prog Brain Res.

[48] Dillon D.G., Pizzagalli D.A. (2018). Mechanisms of memory disruption in depression. Trends Neurosci.

